# Snow white

**DOI:** 10.1007/s12471-017-1017-9

**Published:** 2017-07-13

**Authors:** H. Lameijer, M. Kwant, M. Doff-Holman

**Affiliations:** Department of Emergency Medicine, University Medical Centre Groningen, University of Groningen, Groningen, The Netherlands

A 56-year-old man, admitted to a forensic psychiatric centre, presented at our emergency department. He had been found indoors in a chair, unconscious (Glasgow coma score 1–1–1), duration unknown, with shallow breathing and symmetrical pinpoint pupils. We suspected opioid intoxication and administered naloxone 0.1 mg twice intravenously, without sufficient effect on consciousness or respiration. We sedated, intubated and mechanically ventilated the patient to protect the airway and provide respiratory support. At our emergency department his respiration was stable, with a pulse of 56 bpm without cardiac murmurs, blood pressure 130/80 mm Hg, and a capillary refill time <2 s. His pupils remained pinpoint and an intravenous needle puncture wound was observed in his left arm. Cerebral computed tomography was performed and excluded intracerebral haemorrhage and basilar thrombosis. An electrocardiogram was performed (Fig. 1). What abnormalities can be observed and what do they suggest? What are the implications for further management of this patient?

**Fig. 1:**
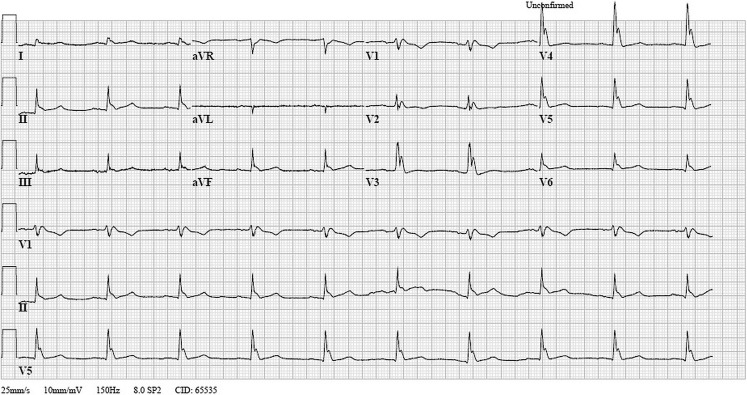
Electrocardogram made after primary survey

## Answer

You will find the answer elsewhere in this issue.

